# Supplementary material for: Moderate beta-cell ablation triggers synergic compensatory mechanisms even in the absence of overt metabolic disruption
Source: Commun Biol. 2024 Jul 9;7:833. doi: 10.1038/s42003-024-06527-5 (PMC11233560; doi:10.1038/s42003-024-06527-5)
Supplement: Supplementary file 2 — Supplementary Information [file 42003_2024_6527_MOESM2_ESM.pdf]

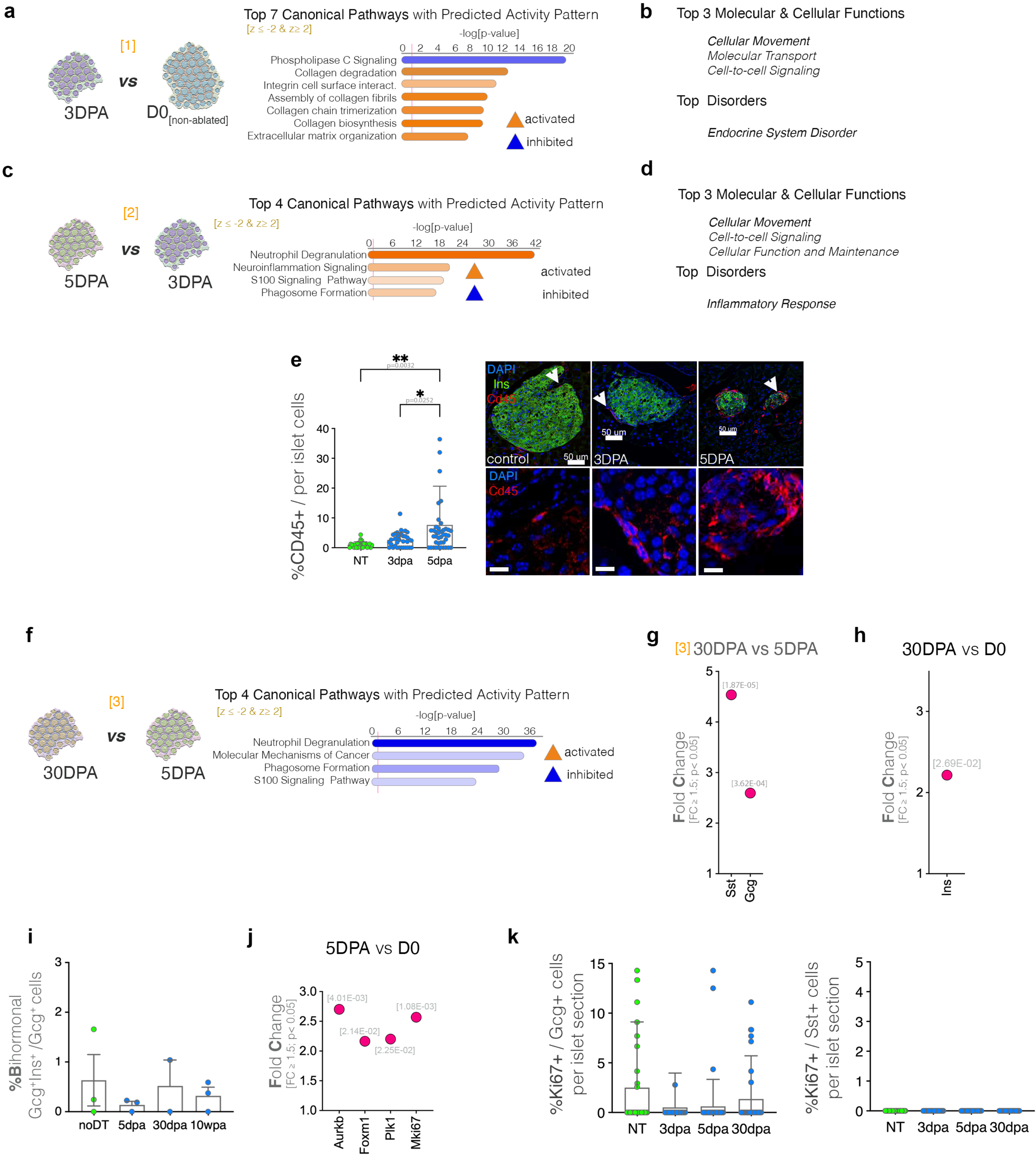

a
Top 7 Canonical Pathways with Predicted Activity Pattern
[z ≤ -2 & z≥ 2]
[1]
3DPA vs D0[non-ablated]
-log[p-value]
Phospholipase C Signaling
Collagen degradation
Integrin cell surface interact.
Assembly of collagen fibrils
Collagen chain trimerization
Collagen biosynthesis
Extracellular matrix organization
activated
inhibited
b
Top 3 Molecular & Cellular Functions
Cellular Movement
Molecular Transport
Cell-to-cell Signaling
Top Disorders
Endocrine System Disorder
c
[2]
5DPA vs 3DPA
Top 4 Canonical Pathways with Predicted Activity Pattern
[z ≤ -2 & z≥ 2]
-log[p-value]
Neutrophil Degranulation
Neuroinflammation Signaling
S100 Signaling Pathway
Phagosome Formation
activated
inhibited
d
Top 3 Molecular & Cellular Functions
Cellular Movement
Cell-to-cell Signaling
Cellular Function and Maintenance
Top Disorders
Inflammatory Response
e
%CD45+ / per islet cells
p=0.0032
p=0.0252
NT
3dpa
5dpa
DAPI
Ins
Cd45
control
3DPA
5DPA
50 um
f
[3]
30DPA vs 5DPA
Top 4 Canonical Pathways with Predicted Activity Pattern
[z ≤ -2 & z≥ 2]
-log[p-value]
Neutrophil Degranulation
Molecular Mechanisms of Cancer
Phagosome Formation
S100 Signaling Pathway
activated
inhibited
g
[3] 30DPA vs 5DPA
Fold Change [FC ≥ 1.5; p< 0.05]
[1.87E-05]
[3.62E-04]
Sst
Gcg
h
30DPA vs D0
[2.69E-02]
Ins
i
%Bihormonal Gcg+Ins+/Gcg+cells
noDT
5dpa
30dpa
10wpa
j
5DPA vs D0
[4.01E-03]
[2.14E-02]
[2.25E-02]
[1.08E-03]
Aurkb
Foxm1
Plk1
Mki67
k
%Ki67+ / Gcg+ cells per islet section
%Ki67+ / Sst+ cells per islet section
NT
3dpa
5dpa
30dpa

Supplemental Figure 1.

(a) Top seven canonical pathways with predicted activity pattern (z-score ≤ -2 [inhibited, blue], z-score ≥ 2 [activated, orange]) defining the transcriptional landscape between control unablated islets and 3 DPA islets. **(b)** List of top three molecular and cellular functions and top disorders category between unablated and 3 DPA islets. **(c)** Top four canonical signaling pathways with predicted activity pattern (z-score ≤ -2 [inhibited, blue], z-score ≥ 2 [activated, orange]) defining the transcriptional landscape between 5 and 3 DPA islets. **(d)** List of top molecular and cellular functions as well as top disorder category differentially regulated between 5 and 3 DPA. **(e)** Representative immunofluorescence images of immune cell markers (scale bar – 50 μm, arrow heads point at the magnified regions, scale bar in zoomed images: 10μm) and respective graphs displaying the quantification of Cd45+ cells per islet section (one-way ANOVA; N=3 mice; an average of 32 islet sections were counted per condition). **(f) T**op four canonical pathways with predicted activity pattern (z-score ≤ -2 [inhibited, blue], z-score ≥ 2 [activated, orange]) defining the transcriptional landscape between 30 and 5 DPA islets. **(g)** Observed upregulation of somatostatin and glucagon between 5 and 30 DPA. **(h)** Observed upregulation of *Ins1* gene when comparing 30 DPA and control unablated islets. **(i)** Graph displaying the quantification of % bihormonal cells (Gcg+/Ins+) from the Gcg+ cell population following moderate DT-induced ablation (one-way ANOVA). **(j)** Observed upregulation of proliferation markers between 5 DPA and control unablated islets. **(k)** Graphs displaying the quantification of Ki67+Gcg+ and Ki67+Sst+ cells per islet section (one-way ANOVA, an average of 59 (for Gcg) and 57 (for Sst) islet sections were counted per condition). Data in (e, i, k) are represented as mean ± SEM.

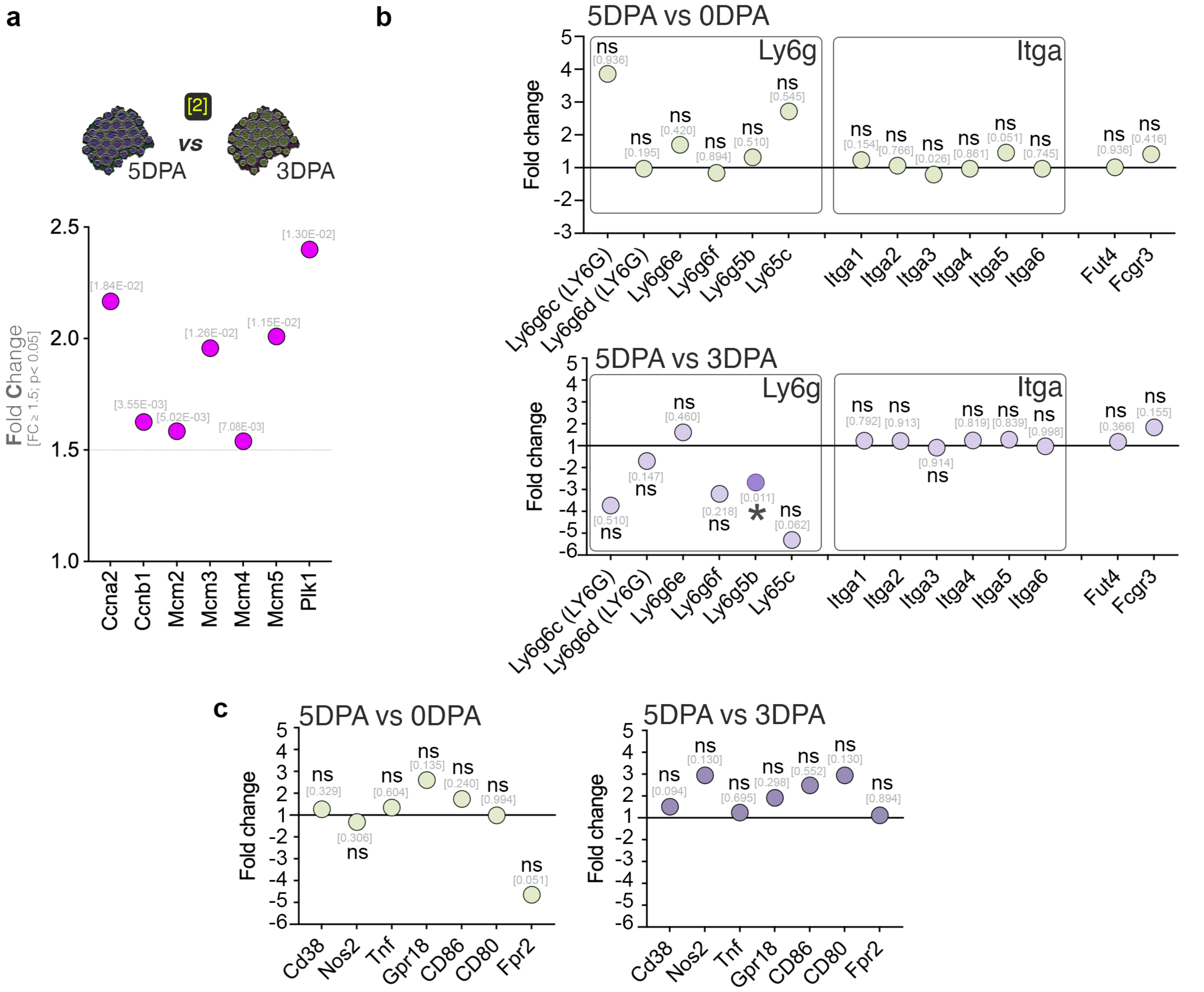

Supplemental Figure 2. (a) Graph depicting the observed upregulation of proliferation markers between 5 and 3 DPA islets of immunodeficient RIP-DTR mice. (b) Graphs depicting the lack of significant regulation of most neutrophile markers at 5 DPA as compared to either control (5 DPA vs CTRL) or 3 DPA (5 DPA vs 3 DPA). (c) Graphs depicting the lack of significant regulation of most M1 macrophage markers at 5 DPA as compared to either control (5 DPA vs CTRL) or 3 DPA (5 DPA vs 3 DPA).
