# Supplementary material for: Moderate beta-cell ablation triggers synergic compensatory mechanisms even in the absence of overt metabolic disruption
Source: Commun Biol. 2024 Jul 9;7:833. doi: 10.1038/s42003-024-06527-5 (PMC11233560; doi:10.1038/s42003-024-06527-5)
Supplement: Supplementary file 3 — Description of Additional Supplementary Files [file 42003_2024_6527_MOESM3_ESM.pdf]

Description of Additional Supplementary Files

File name: Supplementary Data 1

Description: DEG lists used for IPA-driven pathway analysis

File name: Supplementary Data 2

Description: The source data behind the graphs in the paper
