# Supplementary material for: Moderate beta-cell ablation triggers synergic compensatory mechanisms even in the absence of overt metabolic disruption
Source: Commun Biol. 2024 Jul 9;7:833. doi: 10.1038/s42003-024-06527-5 (PMC11233560; doi:10.1038/s42003-024-06527-5)
Supplement: Supplementary file 6 — Reporting Summary [file 42003_2024_6527_MOESM6_ESM.pdf]

Corresponding author(s): *Double-anonymous peer review submissions: write DAPR and your manuscript number here instead of author names.*

Last updated by author(s): *YYYY-MM-DD*

Reporting Summary

Nature Portfolio wishes to improve the reproducibility of the work that we publish. This form provides structure for consistency and transparency in reporting. For further information on Nature Portfolio policies, see our Editorial Policies and the Editorial Policy Checklist.

Statistics

For all statistical analyses, confirm that the following items are present in the figure legend, table legend, main text, or Methods section.

n/a	Confirmed	
☐	☒	The exact sample size (*n*) for each experimental group/condition, given as a discrete number and unit of measurement
☐	☒	A statement on whether measurements were taken from distinct samples or whether the same sample was measured repeatedly
☐	☒	The statistical test(s) used AND whether they are one- or two-sided<br>*Only common tests should be described solely by name; describe more complex techniques in the Methods section.*
☒	☐	A description of all covariates tested
☐	☒	A description of any assumptions or corrections, such as tests of normality and adjustment for multiple comparisons
☐	☒	A full description of the statistical parameters including central tendency (e.g. means) or other basic estimates (e.g. regression coefficient) AND variation (e.g. standard deviation) or associated estimates of uncertainty (e.g. confidence intervals)
☐	☒	For null hypothesis testing, the test statistic (e.g. *F*, *t*, *r*) with confidence intervals, effect sizes, degrees of freedom and *P* value noted<br>*Give P values as exact values whenever suitable.*
☒	☐	For Bayesian analysis, information on the choice of priors and Markov chain Monte Carlo settings
☐	☒	For hierarchical and complex designs, identification of the appropriate level for tests and full reporting of outcomes
☒	☐	Estimates of effect sizes (e.g. Cohen's *d*, Pearson's *r*), indicating how they were calculated

Our web collection on statistics for biologists contains articles on many of the points above.

Software and code

Policy information about availability of computer code

Data collection	automated cell counting macro using ImageJ/Fiji
Data analysis	Insulin volume was quantified using Imaris software (Bitplane), version 9.1.2 (surface segmentation algorithm); RNAseq was analysed using CLC Genomics Workbench (Qiagen), while pathways analyses were performed on Ingenuity Pathway Analysis (IPA®, Qiagen); Venn clustering of differentially expressed genes were performed with Venny version 2.1 (BioinfoGP); statistical analyses were performed using GraphPad Prism v9.5.1 (GraphPad Software Inc., USA).

For manuscripts utilizing custom algorithms or software that are central to the research but not yet described in published literature, software must be made available to editors and reviewers. We strongly encourage code deposition in a community repository (e.g. GitHub). See the Nature Portfolio guidelines for submitting code & software for further information.

Data

Policy information about availability of data

All manuscripts must include a data availability statement. This statement should provide the following information, where applicable:

- Accession codes, unique identifiers, or web links for publicly available datasets
- A description of any restrictions on data availability
- For clinical datasets or third party data, please ensure that the statement adheres to our policy

The data files were uploaded to the GEO database and is available under to following identifiers GSE26517 and GSE26518.

Research involving human participants, their data, or biological material

Policy information about studies with human participants or human data. See also policy information about sex, gender (identity/presentation), and sexual orientation and race, ethnicity and racism.

Reporting on sex and gender	n/a
Reporting on race, ethnicity, or other socially relevant groupings	n/a
Population characteristics	n/a
Recruitment	n/a
Ethics oversight	n/a

Note that full information on the approval of the study protocol must also be provided in the manuscript.

Field-specific reporting

Please select the one below that is the best fit for your research. If you are not sure, read the appropriate sections before making your selection.

☒ Life sciences ☐ Behavioural & social sciences ☐ Ecological, evolutionary & environmental sciences

For a reference copy of the document with all sections, see nature.com/documents/nr-reporting-summary-flat.pdf

Life sciences study design

All studies must disclose on these points even when the disclosure is negative.

Sample size	A minimal sample size of n>3 was used to draw conclusions. Information on sample size is included in corresponding figure legends.
Data exclusions	none
Replication	Biological replicates were included in this study.
Randomization	Random allocation of siblings with the required genotype in the experimental groups.
Blinding	No blinding.

Reporting for specific materials, systems and methods

We require information from authors about some types of materials, experimental systems and methods used in many studies. Here, indicate whether each material, system or method listed is relevant to your study. If you are not sure if a list item applies to your research, read the appropriate section before selecting a response.

Materials & experimental systems

n/a	Involved in the study	
☐	☒	Antibodies
☒	☐	Eukaryotic cell lines
☒	☐	Palaeontology and archaeology
☐	☒	Animals and other organisms
☒	☐	Clinical data
☒	☐	Dual use research of concern
☒	☐	Plants

Methods

n/a	Involved in the study	
☒	☐	ChIP-seq
☒	☐	Flow cytometry
☒	☐	MRI-based neuroimaging

Antibodies

Antibodies used	Guinea Pig anti-insulin (1:400, AE80452 Geneva Antibody Facility), mouse IgG1 anti-glucagon (1:1000, G265453 Sigma-Aldrich), mouse IgG anti-Ki67 (1:1000, ab27965354 Abcam), rabbit anti-somatostatin 2855 (1:400, ab111912 Abcam), chicken anti-insulin (LS-c96116, LSBio), guinea pig anti-glucagon (1:200, AK24756 Geneva Antibody Facility), chicken anti-somatostatin 28 (1:400, 36600657 Synaptic Systems), rabbit anti-insulin (1:200, 15848-1-AP58 Thermo Scientific) and rat anti-CD45 (1:100, NB100-77417, Novus

Biologicals).
Visualisation of the primary stains was done with the following secondary antibodies from Invitrogen: Goat anti-guinea pig A488(A11073), donkey anti-rabbit A546(A10040), Goat anti-chicken A594(A11042), goat anti-chicken A488(A11039), goat anti-mouse A647(A21235), goat anti-mouse IgG1 A647(A21240). All the secondary antibodies, along with nuclear staining with DAPI was done at a concentration of 1:500.

Validation	Use of negative and positive controls. All antibodies used were previously published by other research groups.

Animals and other research organisms

Policy information about studies involving animals; ARRIVE guidelines recommended for reporting animal research, and Sex and Gender in Research

Laboratory animals	female mice of an age younger than 20-weeks
Wild animals	n/a
Reporting on sex	The study used female mice of an age younger than 20-weeks at the time of injections. The choice of all female mice was made to replicate findings in the Hemi-DT model, which can only use females due to the reliance on random X inactivation.
Field-collected samples	n/a
Ethics oversight	Ethical autoriuation was obtain from the Norwegian Animal Research authority and in accordance with the European Union (EU) Directive 2010/63/EU, under the following FOTS licenses: 19800, 25526, 25531, and by the Romanian competent authority under Authorization No. 590/13.01.2021

Note that full information on the approval of the study protocol must also be provided in the manuscript.

Plants

Seed stocks	n/a
Novel plant genotypes	n/a
Authentication	n/a
